# HPV-Driven Immune Evasion in Cervical Cancer: Transcriptomic Identification of Downregulated Hub Genes and Suppressed Leukocyte Migration Pathways

**DOI:** 10.3390/ijms262412121

**Published:** 2025-12-17

**Authors:** Sana Ismael Ameen, Mahla Masoudi, Hossein Azizi, Thomas Skutella

**Affiliations:** 1 Department of Gynecology and Obstetrics, Faculty of General Medicine, Koya University, Koya 44023, Kurdistan Region, Iraq; sana.ismael@koyauniversity.org; 2 Department of Stem Cells and Cancer, College of Biotechnology, Amol University of Special Modern Technologies, Amol 49767, Iran; mahlamasoudii@gmail.com; 3 Institute for Anatomy and Cell Biology, Medical Faculty, University of Heidelberg, Im Neuenheimer Feld 307, 69120 Heidelberg, Germany; thomas.skutella@uni-heidelberg.de

**Keywords:** HPV-driven cervical cancer, immune evasion, transcriptomic analysis, leukocyte transendothelial migration pathway, immune modulation

## Abstract

Cervical cancer progression, particularly in the context of HPV infection, is driven by complex transcriptional alterations within the tumor microenvironment. Understanding the molecular mechanisms underlying HPV-induced immune evasion is crucial for developing effective therapeutic strategies. Transcriptomic analyses were performed using three independent datasets (GSE127265, GSE166466, and GSE218460) to identify differentially expressed genes (DEGs) between HPV-positive and HPV-negative cervical cancer samples. Protein–protein interaction networks were constructed using Cytoscape and STRING, and immune infiltration was assessed via the TIMER database. A total of 572 DEGs were commonly identified between tumor and normal tissues, with HPV-positive samples showing distinct transcriptional profiles. Several downregulated hub genes were associated with immune regulation and receptor tyrosine kinase signaling. Immune infiltration analysis revealed altered dendritic cell and T cell patterns, indicating HPV-mediated immune modulation. Pathway enrichment identified the leukocyte transendothelial migration pathway as a key mechanism impaired by HPV infection. These findings highlight the critical role of immune-related hub genes in HPV-driven cervical cancer progression and suggest potential therapeutic targets to counteract HPV-induced immune suppression.

## 1. Introduction

Cervical cancer is a widespread disease in women, responsible for significant global morbidity and mortality. Among the various risk factors, HPV infection is the leading cause, being implicated in approximately 99.7% of all cases [1].

The human immune system plays a pivotal role in defending against infections, including those caused by HPV. However, in the context of HPV infection, certain immune responses can inadvertently contribute to tumor progression. For instance, an increase in neutrophil populations has been observed to suppress T-cell-mediated antitumor activity, thereby facilitating the development of malignancies and impairing overall immune function [2]. HPV-infected cells also exhibit elevated expression of genes associated with pro-inflammatory mediators, such as interleukin-1 beta (*IL-1β*). The inflammasome, a multi-protein complex, is responsible for the activation of caspase-1, which in turn processes and secretes *IL-1β* and *IL-18*. These cytokines play crucial roles in initiating and propagating inflammatory responses [3]. On the other hand, the *NLRP3* inflammasome acts as a molecular sensor, triggering pyroptosis, which can both promote and hinder cancer progression depending on the tumor microenvironment. Activation of *NLRP3* can enhance immune responses by recruiting immune cells to the tumor, but chronic activation leads to sustained inflammation, promoting tumor growth, invasion, and metastasis. This dual role highlights *NLRP3* as a potential therapeutic target in cancer, as well as its relevance in immune modulation during viral infections like SARS-CoV-2 [4].

Despite significant advances in the study and treatment of cervical cancer caused by HPV infection, there are still considerable gaps in understanding the precise mechanisms involved in the interactions between the virus and the immune system. Recent studies have demonstrated the importance of metabolic and immune-related gene signatures in cancer progression and treatment response. For example, a 12-gene signature related to OXPHOS (oxidative phosphorylation) has been shown to predict ovarian cancer patients’ response to immune checkpoint inhibitors, linking metabolic pathways with immune regulation in cancer [5]. Additionally, the role of exosomal miRNAs in modulating immune responses and inflammation further highlights the complexity of immune evasion mechanisms in cancers, including cervical cancer [6].

A recent transcriptomic study revealed that *EGCG* and *myricetin* exhibited synergistic antiproliferative effects on cervical cancer cell lines (ME180 and SiHa), with strong molecular binding to the *HK2* and *MAP7* targets and associated regulatory miRNAs [7]. Furthermore, another study reported a lectin protein (AEL) derived from Abelmoschus esculentus, which was fully sequenced and functionally characterized. This protein demonstrated selective cytotoxicity against cervical (HeLa) and colon (T84) cancer cell lines, while showing minimal effects on normal HEK293 cells [8].

One of the main challenges is the complexity of the TME, which includes intricate interactions between tumor and immune cells. Recent studies have shown that tumor-associated fibroblasts (CAFs) play a crucial role in cancer progression and resistance to treatments, but the diversity of these cells and their interactions with other immune cells have not been fully elucidated [9]. In addition, our incomplete understanding of immune pathway activation and its regulation in response to HPV infection, particularly at different stages of infection, requires further research. Pathways such as AIM2, which are activated in the early stages of infection, are suppressed in later stages of HPV infection, reflecting the complexity of the virus–immune system interactions [10]. Furthermore, HPV exploits oncogenes like E6 and E7 to challenge the immune system by inhibiting various immune pathways, such as the cGAS-STING pathway, ultimately leading to reduced immune and inflammatory responses [11]. Understanding these mechanisms and examining their impact on the progression of cervical cancer, particularly in advanced stages, remains critically important and necessitates further in-depth investigation. These scientific gaps highlight the need for additional studies on various immune mechanisms and molecular pathways in order to gain a better understanding of HPV-driven cervical cancer development and design more effective therapeutic strategies.

The primary objective of this study is to accurately identify and analyze the role of immune hub genes and molecular pathways in cervical cancer caused by HPV infection. This research aims to examine the complex and precise effects of HPV on the transcriptomic changes and immune responses within the TME using transcriptomic data and advanced analyses. The main innovation of this study lies in focusing on identifying immune hub genes that play crucial roles in processes such as leukocyte migration and immune regulation, which are affected by HPV infection. Additionally, by utilizing protein–protein interaction networks and pathway analyses, the relationship between genetic changes and immune impacts in cervical cancer is comprehensively examined. This research identifies and investigates immune pathways influenced by HPV, revealing their effects on tumor development at both the molecular and cellular levels. These innovations could pave the way for the design of new, targeted therapeutic strategies to combat HPV-related cervical cancer, contributing to more effective prevention and treatment approaches for the disease.

## 2. Results

**Figure 1:**
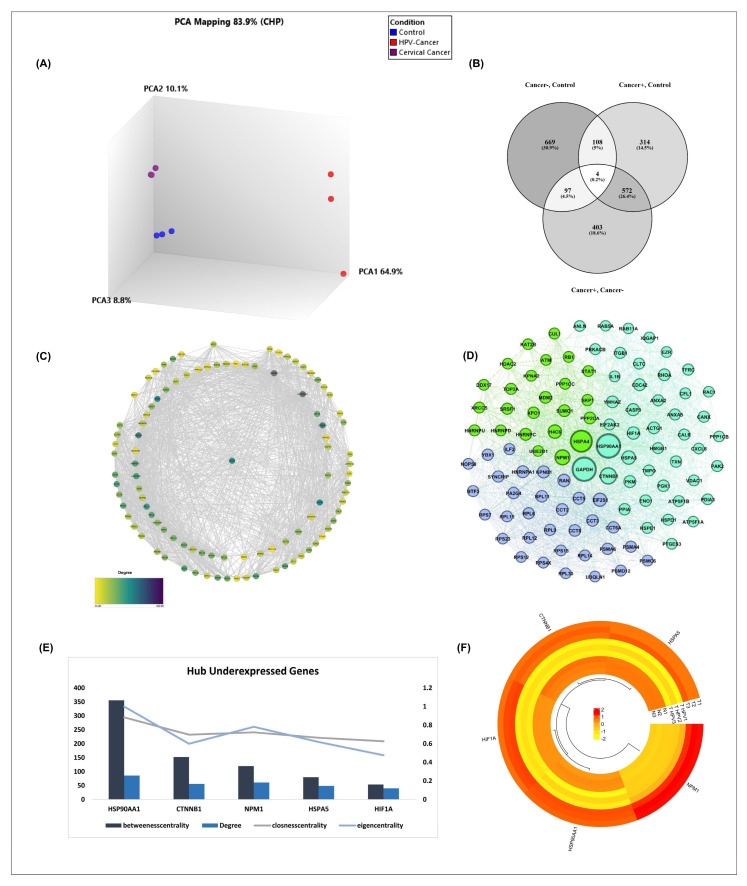
Transcriptomic and Network Analysis of HPV-Associated Cervical Cancer. (**A**) Principal Component Analysis (PCA) illustrating global transcriptional divergence among control, HPV-positive, and HPV-negative cervical cancer samples, accounting for 83.9% of total variance; (**B**) Venn diagram showing differentially expressed gene (DEG) overlaps across three comparisons, highlighting HPV-specific and shared oncogenic signatures; (**C**) PPI network constructed from HPV-specific DEGs using STRING and Cytoscape; node color represents degree centrality; (**D**) Subnetwork of immune- and RTK-related genes identified through centrality-based filtering; (**E**) Bar graph showing top 5 underexpressed hub genes ranked by centrality measures; (**F**) Heatmap indicating reduced expression of selected hub genes in HPV-positive samples, supporting virus-mediated transcriptional repression.

Transcriptional alterations linked to cervical cancer and HPV infection were investigated through comparative analysis of three independent datasets: GSE127265 (cervical cancer), GSE166466 (HPV-positive cervical cancer), and GSE227550 (normal cervical tissues). Following normalization and differential expression analysis, principal component analysis (PCA) was performed to evaluate the global transcriptional variation among the three datasets. As shown in Figure 1A, the first three principal components accounted for 83.9% of the total variance (PC1: 64.9%, PC2: 10.1%, PC3: 8.8%). The PCA plot revealed a clear separation between cancer samples and normal controls, indicating robust transcriptional differences associated with malignant transformation. Moreover, HPV-positive and HPV-negative cervical cancers exhibited partial divergence in their expression profiles, suggesting HPV-dependent modulation of gene expression in addition to cancer-related alterations.

To identify DEGs and their overlaps between groups, a Venn diagram was generated (Figure 1B). Comparative analysis revealed 669 DEGs unique to HPV-negative cervical cancer compared with controls, whereas 314 genes were uniquely dysregulated in HPV-positive cervical cancers. A total of 572 genes were commonly deregulated between both cancer groups relative to normal tissues, representing a shared oncogenic transcriptional signature. Interestingly, 108 genes were shared between HPV-negative cancers and HPV-positive cancers, while 97 genes were unique to the HPV-negative versus HPV-positive comparison. Only four genes were consistently altered across all three datasets (Detailed results are provided in Supplementary Files S1–S3). These findings indicate both shared and HPV-specific transcriptional programs in cervical cancer progression. To further delineate HPV-specific transcriptional alterations, we focused on genes that were uniquely dysregulated between HPV-positive and HPV-negative cervical cancers. These gene subsets represent molecular differences potentially attributable to viral oncogenic activity rather than the malignant process per se.

Molecular interactions underlying HPV-associated transcriptional changes were explored by constructing a PPI network using Cytoscape (Figure 1C). Application of stringent filtering criteria (adjusted *p* < 0.05 and |log2FC| ≥ 2) reduced the candidate gene set to 100 hub genes with the highest degree of connectivity. Notably, all hub genes identified in this analysis were downregulated, indicating that HPV infection is associated with a global repression of central regulatory genes within the cervical cancer transcriptome. In the PPI network, node color intensity corresponded to the degree parameter, highlighting highly connected genes that may act as key regulators (Figure 1D).

Gephi software (v0.9.2) was utilized for advanced network topology analysis, enabling further characterization of the functional relevance of identified hub genes, with a specific focus on immune-related genes and RTKs. Network clustering based on centrality measures (degree, betweenness, closeness, and eigenvector) enabled the identification of the most influential hub genes (Figure 1E). These genes, predominantly associated with immune surveillance and RTK signaling, emerged as potential key drivers in HPV-positive cervical cancer.

Heatmap visualization further demonstrated the expression patterns of the top-ranked hub genes (Figure 1F). Consistent with the PPI and centrality analyses, these genes displayed a marked reduction in expression in HPV-positive samples compared with HPV-negative cancers and normal tissues. The uniform downregulation of immune-related and RTK-associated hub genes in the presence of HPV strongly suggests a virus-mediated suppression of tumor immune responsiveness and tyrosine kinase signaling, which may have implications for both disease progression and therapeutic intervention.

The impact of copy number alterations (CNAs) in the identified hub genes on the tumor immune microenvironment was evaluated by analyzing their association with immune cell infiltration levels in CESC using the TIMER database. The results revealed distinct patterns of immune modulation depending on the specific gene and CNA status.

**Figure 2:**
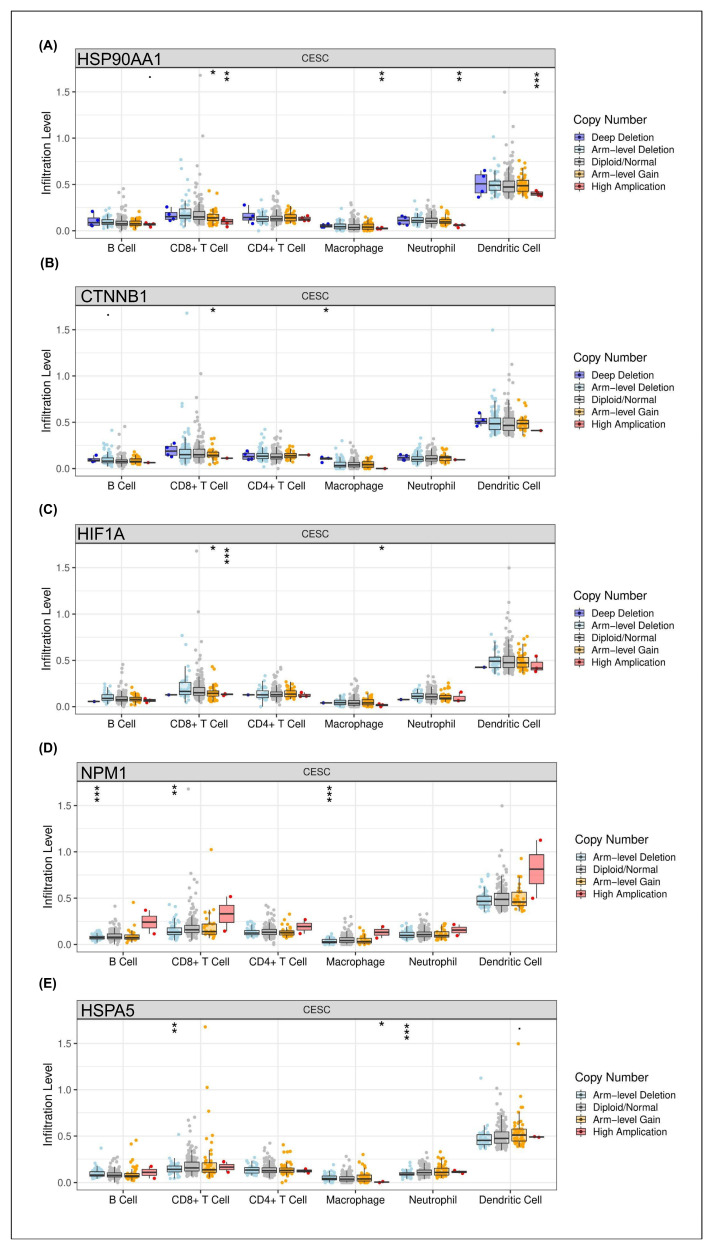
Immune Infiltration Patterns Associated with CNVs in Hub Genes. Boxplots of immune cell infiltration stratified by copy number variation (CNV) for five hub genes: (**A**) *HSP90AA1*; (**B**) *CTNNB1*; (**C**) *HIF1A*; (**D**) *NPM1*; and (**E**) *HSPA5*, across B cells, CD4^+^/CD8^+^ T cells, macrophages, neutrophils, and dendritic cells. Significant correlations are observed between gene amplification and dendritic/CD8^+^ T cell infiltration, highlighting immune-modulatory consequences of CNV alterations in HPV-driven cervical cancer. (*p*-value significant codes: 0 ≤ *** < 0.001 ≤ ** < 0.01 ≤ * < 0.05 ≤ < 0.1) (*p*-value significance codes: *** ≤ 0.001, ** ≤ 0.01, * ≤ 0.05, and *p*-value < 0.1). Each dot represents an individual data point for different cell types (e.g., B Cell, CD8^+^ T Cell, etc.) categorized by their respective copy numbers.

Specifically, a significant association between *HSPA90AA1* amplification and increased dendritic cell infiltration was observed, whereas relatively lower immune cell levels were found in diploid/normal and deletion states (Figure 2A). Similarly, strong correlations between *CTNNB1* CNAs and dendritic cell recruitment were detected, suggesting that antigen presentation capacity in CESC may be influenced by alterations in Wnt/β-catenin signaling (Figure 2B). For *HIF1A*, known as a hypoxia-inducible transcription factor, copy number variation was modestly linked to neutrophil and dendritic cell infiltration, indicating partial regulation of innate immune responses within the tumor microenvironment by hypoxic signaling (Figure 2C). In contrast, significant correlations between *NPM1* alterations and changes in B cell and CD8^+^ T cell infiltration were identified, implicating this nucleolar protein in adaptive immune modulation (Figure 2D). Interestingly, consistent associations between *HSPA5* CNAs and dendritic cell as well as CD8^+^ T cell infiltration were observed (Figure 2E).

**Figure 3:**
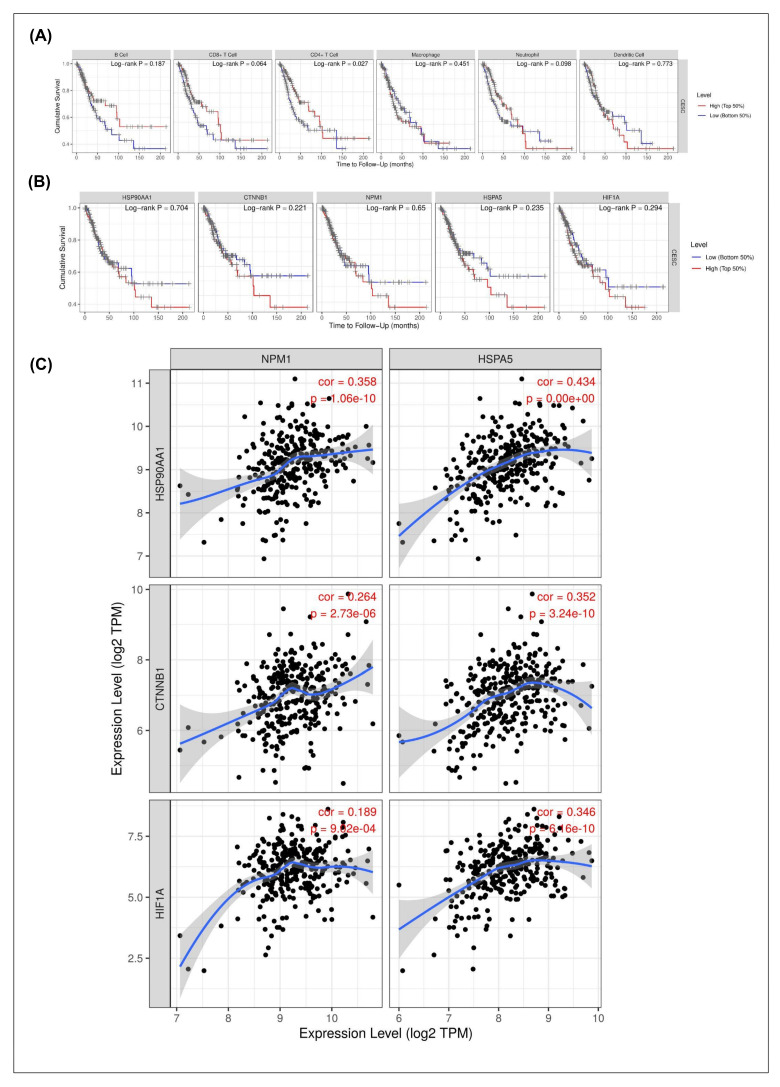
Prognostic and Co-expression Analysis of Immune-Related Hub Genes. (**A**) Kaplan–Meier survival curves assessing correlation between immune cell infiltration levels and overall survival; only CD4^+^ T cell infiltration shows significant prognostic value (*p* = 0.027); (**B**) Survival analysis of hub gene expression shows no statistically significant association with survival, suggesting non-prognostic expression profiles; (**C**) Co-expression plots demonstrating significant positive correlations between *HSP90AA1* and *NPM1*/*HSPA5*, and moderate correlations among other hub genes, indicating interconnected transcriptional regulation among immune and stress response genes.

The prognostic relevance of immune infiltration and hub gene expression in CESC was assessed using Kaplan–Meier survival analyses (Figure 3A,B). Among immune cell subsets, only CD4^+^ T cell infiltration showed a significant association with patient survival (log-rank *p* = 0.027), whereas B cells, CD8^+^ T cells, macrophages, neutrophils, and dendritic cells did not display statistically significant effects (Figure 3A). This finding suggests that CD4^+^ T cells may play a more prominent role in shaping clinical outcomes compared to other immune populations in CESC.

With respect to hub genes, none of the five candidates (*HSPA90AA1*, *CTNNB1*, *NPM1*, *HSPA5*, and *HIF1A*) demonstrated a statistically significant correlation with overall survival in the TCGA-CESC cohort (all log-rank *p* > 0.05; Figure 3B). These results indicate that although these genes are differentially expressed and immune-associated, their transcriptomic levels alone may not serve as robust independent prognostic markers in cervical cancer.

Co-expression correlation analysis was conducted to further explore the molecular interrelationships among the hub genes (Figure 3C). *NPM1* and *HSPA5* both showed a strong positive correlation with *HSPA90AA1* expression (r = 0.358 and r = 0.434, respectively; *p* < 1.0 × 10^−10^), suggesting coordinated regulation of stress-related chaperone pathways. In contrast, *CTNNB1* exhibited a weak negative correlation with *NPM1* (r = −0.264; *p* = 2.73 × 10^−6^) but a positive correlation with *HSPA5* (r = 0.352; *p* = 3.24 × 10^−10^), highlighting divergent regulatory dynamics within the Wnt/β-catenin signaling context. Similarly, *HIF1A* expression was moderately correlated with both *NPM1* (r = 0.189; *p* = 8.92 × 10^−4^) and *HSPA5* (r = 0.346; *p* = 6.6 × 10^−10^), consistent with hypoxia-driven stress response networks.

**Figure 4:**
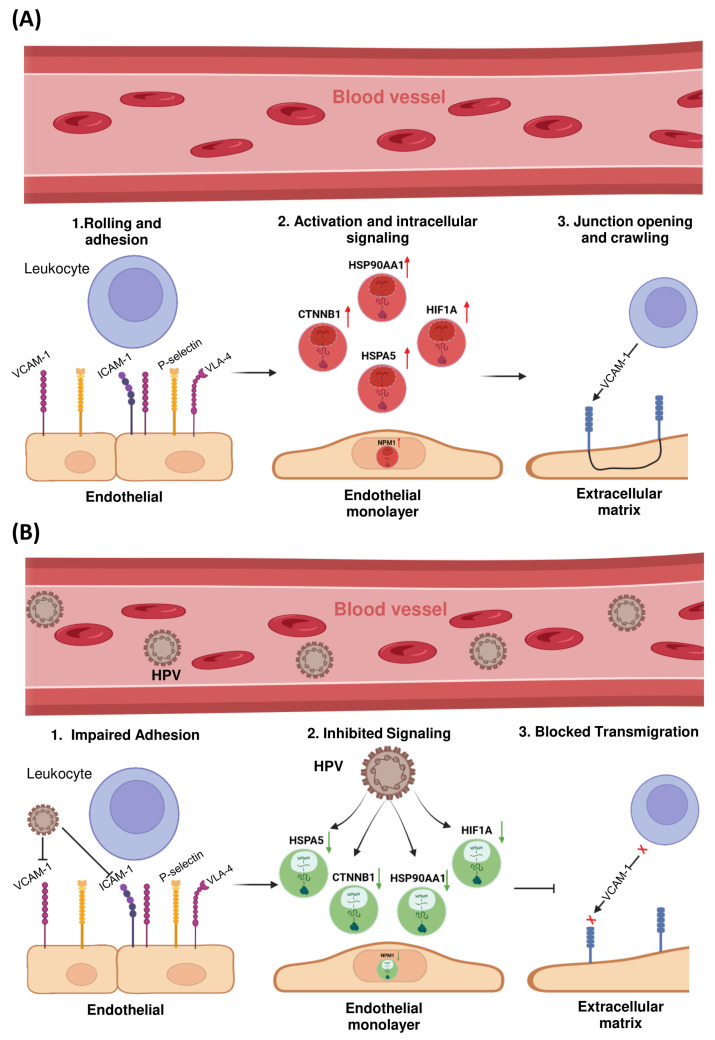
Schematic of Leukocyte Transendothelial Migration in Normal and HPV+ Context. (**A**) Normal LTM pathway showing leukocyte adhesion, activation, and transmigration via VCAM-1/ICAM-1 signaling and hub gene participation (*HSP90AA1*, *CTNNB1*, *HIF1A*, *HSPA5*, *NPM1*); (**B**) In HPV-positive context, viral activity suppresses expression of LTM-related hub genes, leading to impaired leukocyte adhesion, disrupted signaling, and reduced transmigration—suggesting a mechanism of immune evasion in cervical cancer. (Figure created by the authors).

Gene Ontology (GO) and Kyoto Encyclopedia of Genes and Genomes (KEGG) enrichment analyses were performed on the top-ranked hub genes—identified through PPI network and centrality analyses—to gain mechanistic insights into the functional implications of HPV-associated transcriptional alterations. As illustrated in Figure 4, chord plots revealed that these hub genes were significantly enriched in multiple oncogenic and immune-related pathways.

Specifically, KEGG pathway annotation highlighted four major biological processes: antigen processing and presentation, HIF-1 signaling pathway, leukocyte transendothelial migration, and general pathways in cancer. These results underscore the interplay between viral modulation of host immune surveillance mechanisms and classical cancer-related signaling cascades. Notably, genes such as *HSPA5*, *CTNNB1*, *HIF1A*, and *NPM1* exhibited cross-pathway involvement, suggesting a multifaceted role in HPV-driven oncogenesis.

In the GO biological process category, top enriched terms included “peptide antigen assembly with MHC class I protein complex” and “MHC class I protein complex assembly”, reinforcing the hypothesis of HPV-mediated immune evasion through impaired antigen presentation. Additional enrichment in endothelial cell adhesion and T-cell mediated immune response pathways points to alterations in tumor–immune microenvironment remodeling.

Within the cellular component ontology, the hub genes were predominantly localized to the β-catenin–TCF complex, focal adhesion sites, and cell–substrate junctions, indicating perturbations in cell adhesion, polarity, and signal transduction hubs—key hallmarks of epithelial-to-mesenchymal transition (EMT) and metastasis.From a molecular function perspective, the most enriched terms were associated with ubiquitin-like protein ligase binding, transcription coactivator binding, and protein kinase inhibitor activity (Table 1). These functions suggest that HPV infection exerts widespread effects on proteostasis, transcriptional regulation, and intracellular signaling balance, potentially contributing to both tumor initiation and progression.

The LTM pathway was prioritized for further investigation due to the consistent enrichment of multiple HPV-associated hub genes within this axis. Given its critical role in regulating immune cell infiltration into the tumor microenvironment—and its previously reported dysregulation in HPV-positive cervical cancers—this pathway constitutes a biologically and clinically relevant focus for understanding mechanisms of viral immune evasion.

To further elucidate the mechanistic involvement of these hub genes in immune modulation, a schematic representation of the LTM pathway was constructed (Figure 4). This visualization mapped the transcriptionally dysregulated genes onto key regulatory nodes within the pathway, revealing potential disruptions in immune cell trafficking across the endothelial barrier.

Notably, *HSPA5*, *CTNNB1*, *HIF1A*, and *NPM1* emerged as central modulators of this axis. *HSPA5*, an endoplasmic reticulum chaperone involved in cellular stress responses, was found to influence the expression and trafficking of adhesion molecules such as ICAM-1 and VCAM-1, potentially impairing leukocyte adhesion and extravasation. Similarly, *CTNNB1* (β-catenin), a critical component of adherens junctions and the Wnt signaling pathway, was associated with modulation of endothelial permeability and tight junction dynamics.

In addition, activation of *HIF1A* under hypoxic conditions may promote endothelial barrier stabilization while simultaneously altering chemokine gradients—together contributing to reduced immune cell infiltration into the tumor microenvironment. *NPM1*, a multifunctional nucleolar protein, was implicated in cytoskeletal remodeling and intracellular signaling crosstalk that governs transendothelial migration.

The observed downregulation of these hub genes in HPV-positive cervical cancer samples suggests that HPV infection may contribute to immune evasion not only by impairing antigen processing, but also by actively inhibiting leukocyte recruitment via suppression of the transendothelial migration pathway.

## 3. Discussion

This study has provided significant insights into the molecular mechanisms underlying immune evasion in HPV-driven cervical cancer. Through the transcriptomic analysis of HPV-positive and HPV-negative cervical cancer datasets, we identified a set of immune-related hub genes whose expression is downregulated in the presence of HPV. Notably, the LTM pathway emerged as a key immune evasion mechanism, highlighting how HPV infection impairs leukocyte adhesion and migration. These findings emphasize the role of specific immune pathways, such as the regulation of dendritic cell and T cell infiltration, in shaping the TME of HPV-positive cervical cancer. Furthermore, while hub gene expression did not correlate with overall survival, their regulatory interactions within the TME suggest a multifaceted role in immune modulation.

Our findings are consistent with previous studies that have highlighted immune evasion as a critical component of HPV-associated cancer progression. For instance, research by Avila et al. demonstrated that HPV infection disrupts immune surveillance through the modulation of pro-inflammatory cytokines and immune cell recruitment [12]. Similarly, Che et al. highlighted that HPV oncoproteins E6 and E7 suppress the function of immune cells, including T-cells and dendritic cells, which are crucial for anti-tumor immunity [13].

In support of regulatory RNA networks in cervical cancer, previous findings have shown that hsa_circ_0000021 and KPNA2 are overexpressed and act as oncogenic drivers by negatively regulating miR-3940-3p. Functional studies demonstrated that silencing either hsa_circ_0000021 or KPNA2 suppressed cervical cancer cell proliferation, invasion, and tumor growth, whereas inhibition of miR-3940-3p enhanced malignancy. Mechanistically, hsa_circ_0000021 promotes cervical cancer progression by sponging miR-3940-3p, which targets KPNA2 [14].

Our study corroborates these findings by revealing the downregulation of immune-related hub genes such as *HSPA90AA1*, *CTNNB1*, and *HIF1A* in HPV-positive cervical cancer, which could impair immune cell infiltration and contribute to immune escape. Moreover, while earlier studies focused on global immune suppression, our analysis provides a deeper understanding of how specific signaling pathways, such as RTK and Wnt/β-catenin, influence immune cell infiltration.

Although the identified immune-related hub genes (*HSP90AA1*, *CTNNB1*, *NPM1*, *HSPA5*, and *HIF1A*) were significantly downregulated in HPV-positive cervical cancer, none of them demonstrated a statistically significant correlation with overall survival in the TCGA-CESC cohort. Several factors may contribute to this lack of prognostic association. First, sample heterogeneity and limited cohort size can lead to underpowered survival analysis, especially when stratifying patients into high- and low-expression groups. Second, these hub genes may exhibit functional redundancy, where other genes compensate for their loss, diminishing their individual impact on clinical outcomes. Third, post-transcriptional regulation, such as changes in protein activity, localization, or degradation, might decouple gene expression levels from functional effects on survival. Finally, survival outcomes in cervical cancer are influenced by multiple variables, including treatment regimens, tumor stage, and immune contexture, which may confound single-gene prognostic signals. These findings highlight that although transcriptomic alterations of immune-related genes provide mechanistic insights into HPV-driven immune evasion, they may not serve as standalone prognostic biomarkers without integration into multi-gene or pathway-based models. Interestingly, our results also contrast with studies that have not observed significant correlations between hub gene expression and survival outcomes. For instance, previous research has identified ZNF695 as an oncogenic factor in CESC, where its overexpression was significantly associated with higher histological grade and poor prognosis, including reduced overall survival, progression-free survival, and disease-specific survival. Moreover, ZNF695 was linked to steroid hormone biosynthesis and immune infiltration, suggesting its potential as a prognostic biomarker and therapeutic target [15]. This difference could be due to the complex, multifactorial nature of immune response regulation in cervical cancer.

The downregulation of immune-related hub genes, particularly those involved in LTM, offers novel mechanistic insights into HPV-mediated immune evasion. The LTM pathway, which regulates immune cell trafficking across the endothelial barrier, is disrupted in HPV-positive cervical cancer due to the reduced expression of key genes such as *HSPA5*, *CTNNB1*, and *NPM1*. *HSPA5*, an ER chaperone, influences the expression of adhesion molecules such as ICAM-1 and VCAM-1, impairing immune cell adhesion and extravasation [16]. Similarly, *CTNNB1*, a core component of the Wnt/β-catenin signaling pathway, affects endothelial permeability, further hindering leukocyte infiltration [17]. Hypoxia-driven activation of *HIF1A* also contributes to immune suppression by altering chemokine gradients and stabilizing the endothelial barrier, thereby reducing immune cell infiltration [18].

These findings suggest that HPV-induced alterations in the immune microenvironment are not limited to immune cell dysfunction but also involve structural changes in the endothelial layer, impairing immune cell trafficking and subsequent immune response.

Collectively, these analyses demonstrate that although neither immune infiltration nor hub gene expression levels predict survival outcomes in CESC, their interconnected regulatory networks and coordinated transcriptional programs suggest functional crosstalk between immune-related and RTK/stress-related signaling pathways. This may provide a mechanistic link between viral oncogenesis, tumor immune evasion, and adaptive stress responses in cervical cancer.

The identification of immune-related hub genes involved in HPV-driven immune evasion provides valuable targets for therapeutic intervention. Targeting key genes in the LTM pathway, such as *HSPA5* and *CTNNB1*, could offer novel approaches to enhance immune cell infiltration and restore immune surveillance in HPV-positive cervical cancer. Additionally, manipulating the Wnt/β-catenin and hypoxia pathways may serve as promising therapeutic strategies to counteract HPV-mediated immune suppression.

Future studies should focus on the functional validation of these hub genes in preclinical models and clinical settings. Further investigation into the role of immune checkpoint inhibitors in restoring immune function in the context of HPV-related cervical cancer is warranted. Moreover, exploring combination therapies that target both immune evasion mechanisms and cancer cell survival pathways could improve treatment outcomes for patients with HPV-positive cervical cancer.

## 4. Materials and Methods

### 4.1. Data Collection

Publicly available transcriptomic datasets were retrieved from the Gene Expression Omnibus (GEO/https://www.ncbi.nlm.nih.gov/geo/) database (NCBI) (accessed on 1 October 2025) for comparative analysis. Three independent datasets representing distinct biological conditions were included: GSE127265 (cervical cancer squamous cell carsinoma; GSM3633371, GSM3633372, GSM3633373), GSE166466 (HPV16-positive cervical squamous cell carcinoma; GSM5071468, GSM5071469, GSM5071472), and GSE218460 (normal cervical tissues; GSM6745573, GSM6745574, GSM6745575). All samples were generated using the Affymetrix Human Clariom D Assay platform, enabling high-resolution transcriptome profiling across coding and non-coding RNAs. Raw microarray data were downloaded in CEL file format, which preserves probe-level intensity measurements. The datasets were selected to enable a systematic evaluation of transcriptional alterations associated with malignant transformation and HPV infection in cervical tissues. All CEL files were uniformly processed through standardized normalization and quality control workflows, as described in the following sections [19].

### 4.2. Data Preprocessing and Normalization

Raw CEL files from all samples were processed using Transcriptome Analysis Console (TAC) version 4.0. Data were generated on the Affymetrix Human Clariom D, and normalized using the Robust Multi-array Average (RMA) method, which includes background correction, quantile normalization, and summarization [20]. Differential gene expression was assessed across three comparisons: Cervical Cancer vs. Control, Cervical Cancer vs. HPV-positive Cancer, and Control vs. HPV-positive Cancer, each involving 3 samples per group. The largest number of DEGs was identified in the Cervical Cancer vs. HPV-positive Cancer comparison (14,377 upregulated, 44,640 downregulated). In total, tens of thousands of DEGs were identified across comparisons, with a subset filtered using *p* ≤ 0.05 and |log2FC| ≥ 4, resulting in a focused list of 1000 DEGs. DEG lists were extracted from TAC outputs, and genes were classified as upregulated and downregulated based on the sign of log2FC. Venn analysis using Venny 2.1 was conducted to identify shared and unique DEGs between groups. Subsequent analyses focused on HPV-specific DEGs to explore immune-related transcriptional changes in the tumor microenvironment [21].

### 4.3. PPI Network Construction

The list of HPV-positive and HPV-negative cervical cancer–specific hub genes was submitted to the STRING (v12.0) (https://string-db.org/, accessed on 1 October 2025) database to construct a PPI network. The interaction data were then imported into Cytoscape (v3.10.1) for topological analysis. To refine the network, stringent filtering was applied based on node-level parameters including degree centrality, betweenness centrality, and closeness centrality, reducing the network to the top 100 hub genes with the highest connectivity [22]. The resulting subnetwork was exported to Gephi (v0.9.2) for advanced network visualization and clustering. Using integrated centrality-based algorithms, hub genes were clustered into distinct modules, revealing functional groupings and key regulatory structures within the network [23].

### 4.4. Immune Infiltration and Prognostic Relevance

To assess the immunological and prognostic associations of HPV-related hub genes, gene-level analyses were performed using the tumor immune estimation resource (TIMER, v2.0) database (https://cistrome.shinyapps.io/timer/, accessed on 1 October 2025). The selected hub genes (*HSPA90AA1*, *CTNNB1*, *HIF1A*, *NPM1*, and *HSPA5*) were submitted to evaluate their relationship with immune cell infiltration and copy number variation (CNV) across CESC samples. CNV-based immune infiltration analysis was conducted for six major immune cell subsets: B cells, CD8^+^ T cells, CD4^+^ T cells, macrophages, neutrophils, and dendritic cells. The data were stratified based on five CNV categories: deep deletion, arm-level deletion, diploid/normal, arm-level gain, and high amplification. In addition, Kaplan–Meier survival analysis was performed to assess the association between gene expression levels and overall survival in the TCGA-CESC cohort. Patients were divided into high- and low-expression groups based on median expression values. Finally, gene co-expression correlation analysis was conducted using log2 TPM-normalized expression data to explore regulatory relationships among the selected hub genes [24].

### 4.5. Functional Enrichment and Pathway Mapping

To identify enriched signaling pathways and biological processes associated with the selected hub genes, functional enrichment analysis was performed using the Enrichr database (https://maayanlab.cloud/Enrichr/, accessed on 1 October 2025). Enrichment results were retrieved for both KEGG pathways and Gene Ontology (GO) terms, including Biological Process (BP), Cellular Component (CC), and Molecular Function (MF) categories. The pathway showing the highest degree of hub gene involvement was selected for further visualization [25]. A schematic representation of this pathway was then constructed using the BioRender (https://biorender.com) platform, incorporating relevant molecular components and regulatory interactions.

## 5. Conclusions

This study provides novel insights into HPV-driven immune evasion in cervical cancer, identifying key immune-related hub genes, including *HSPA5*, *CTNNB1*, and *NPM1*, which are downregulated in HPV-positive tumors. These genes play crucial roles in immune cell infiltration and tumor progression through pathways like leukocyte transendothelial migration. Our findings suggest that targeting these pathways, particularly Wnt/β-catenin and hypoxia signaling, may offer new therapeutic strategies for combating HPV-related immune suppression. Although no direct correlation was found between hub gene expression and survival, these genes could serve as potential biomarkers for immune modulation. Further functional validation and clinical trials are needed to explore their therapeutic potential, offering a foundation for more targeted, effective treatments in HPV-associated cervical cancer.

## 6. Limitations and Future Directions

This study provides valuable insights into the immune evasion mechanisms in HPV-driven cervical cancer; however, there are several limitations that need to be acknowledged. First, the analysis was based on publicly available transcriptomic datasets, which may limit the ability to capture the full complexity of the tumor microenvironment in vivo. Due to platform compatibility constraints, all transcriptomic data were limited to the Affymetrix Human Clariom D Assay platform. As a result, the number of available samples per group was restricted to three, which may limit statistical power. Nevertheless, this uniformity minimized batch effects and allowed consistent cross-group comparison. Although we identified key hub genes involved in immune modulation, the functional validation of these genes in experimental models or clinical samples is still lacking. Additionally, while we focused on HPV16-positive and HPV16-negative cancer subtypes, other factors such as HPV variants or patient-specific immune responses were not explored, which may influence the generalizability of our findings. Lastly, the survival analysis did not reveal significant prognostic value for the identified hub genes, suggesting the need for more in-depth investigation into the functional roles of these genes within the broader context of cervical cancer progression. Future studies should aim to validate the role of the identified hub genes in immune modulation through in vitro and in vivo experiments, utilizing patient-derived samples to confirm their relevance in clinical settings. Additionally, exploring the impact of HPV variants on immune evasion mechanisms could provide further insight into the virus–host interaction and its effect on tumor progression. Investigating the potential of combining immunotherapies targeting these hub genes with traditional treatments, such as chemotherapy and radiation, could open new avenues for enhancing therapeutic efficacy. Moreover, more comprehensive immune profiling of the tumor microenvironment, including immune cell subtypes and immune checkpoint regulators, will be crucial to better understand the complex immune landscape in HPV-driven cervical cancer and identify additional therapeutic targets.

## Figures and Tables

**Table 1:** Enriched Pathways and Functional Terms Based on Hub Gene Analysis.

Enrichment	Term	Combined Score
KEGG	Antigen processing and presentation	222.7961
KEGG	Leukocyte transendothelial migration	182.2406
KEGG	HIF-1 signaling pathway	130.71
KEGG	Pathways in cancer	83.08363
BP	Peptide Antigen Assembly With MHC Class I Protein Complex	9808.375163
BP	MHC Class I Protein Complex Assembly	9808.375163
BP	Regulation of Cell Adhesion Molecule Production	7103.106518
BP	Positive Regulation of Vascular Endothelial Growth Factor Receptor Signaling Pathway	2796.457645
BP	Regulation of T-Cell-Mediated Immune Response to Tumor Cell	2471.302025
CC	beta-catenin-TCF Complex	542.7073704
CC	Focal Adhesion	215.1642855
CC	Cell–Substrate Junction	208.7623811
CC	Intracellular Organelle Lumen	157.1103577
CC	Nucleus	20.99353281
MF	Ubiquitin-Like Protein Ligase Binding	888.2403917
MF	Ubiquitin Protein Ligase Binding	611.062523
MF	Transcription Coregulator Binding	365.3276764
MF	DNA-binding Transcription Factor Binding	82.9486113
MF	Protein Kinase Inhibitor Activity	61.21097904

## Data Availability

The datasets generated during and/or analyzed during the current study are available in the GSE datasets repository, [https://www.ncbi.nlm.nih.gov/geo/query/acc.cgi?acc=GSE127265 (accessed on 1 October 2025); https://www.ncbi.nlm.nih.gov/geo/query/acc.cgi?acc=GSE166466 (accessed on 1 October 2025); https://www.ncbi.nlm.nih.gov/geo/query/acc.cgi?acc=GSE227550 (accessed on 1 October 2025)].

## Supplementary Materials

The following supporting information can be downloaded at: https://www.mdpi.com/article/10.3390/ijms262412121/s1.

## Abbreviations

HPV	Human Papillomavirus
CESC	Cervical Squamous Cell Carcinoma
DEGs	Differentially Expressed Genes
PPI	Protein–Protein Interaction
RTK	Receptor Tyrosine Kinase
LTM	Leukocyte Transendothelial Migration
TME	Tumor Microenvironment
